# Food-borne norovirus-outbreak at a military base, Germany, 2009

**DOI:** 10.1186/1471-2334-10-30

**Published:** 2010-02-17

**Authors:** Maria Wadl, Kathrin Scherer, Stine Nielsen, Sabine Diedrich, Lüppo Ellerbroek, Christina Frank, Renate Gatzer, Marina Hoehne, Reimar Johne, Günter Klein, Judith Koch, Jörg Schulenburg, Uta Thielbein, Klaus Stark, Helen Bernard

**Affiliations:** 1 Postgraduate Training for Applied Epidemiology (PAE), Germany; 2 Robert Koch Institute, Berlin, Germany; 3 University of Veterinary Medicine Hannover, Germany; 4 European Programme for Intervention Epidemiology Training (EPIET), European Centre for Disease Prevention and Control, Stockholm, Sweden; 5 Federal Institute for Risk Assessment, Berlin, Germany; 6 Central Institute of the Bundeswehr Medical Service, Berlin, Kiel, Germany; 7 Bundeswehr School of Dog Handling, Ulmen, Germany; 8 Major Medical Clinic with Specialty Services, Leipzig, Germany

## Abstract

**Background:**

Norovirus is often transmitted from person-to-person. Transmission may also be food-borne, but only few norovirus outbreak investigations have identified food items as likely vehicles of norovirus transmission through an analytical epidemiological study.

During 7-9 January, 2009, 36 persons at a military base in Germany fell ill with acute gastroenteritis. Food from the military base's canteen was suspected as vehicle of infection, norovirus as the pathogen causing the illnesses. An investigation was initiated to describe the outbreak's extent, to verify the pathogen, and to identify modes of transmission and source of infection to prevent further cases.

**Methods:**

For descriptive analysis, ill persons were defined as members of the military base with acute onset of diarrhoea or vomiting between 24 December 2008, and 3 February 2009, without detection of a pathogen other than norovirus in stools. We conducted a retrospective cohort study within the headquarters company. Cases were military base members with onset of diarrhoea or vomiting during 5-9 January. We collected information on demographics, food items eaten at the canteen and contact to ill persons or vomit, using a self-administered questionnaire. We compared attack rates (AR) in exposed and unexposed persons, using bivariable and multivariable logistic regression modelling. Stool specimens of ill persons and canteen employees, canteen food served during 5-7 January and environmental swabs were investigated by laboratory analysis.

**Results:**

Overall, 101/815 (AR 12.4%) persons fell ill between 24 December 2008 and 3 February 2009. None were canteen employees. Most persons (n = 49) had disease onset during 7-9 January. Ill persons were a median of 22 years old, 92.9% were male. The response for the cohort study was 178/274 (72.1%). Of 27 cases (AR 15.2%), 25 had eaten at the canteen and 21 had consumed salad. Salad consumption on 6 January (aOR: 8.1; 95%CI: 1.5-45.4) and 7 January (aOR: 15.7; 95%CI: 2.2-74.1) were independently associated with increased risk of disease.

Norovirus was detected in 8/28 ill persons' and 4/25 canteen employees' stools, 6/55 environmental swabs and 0/33 food items. Sequences were identical in environmental and stool samples (subtype II.4 2006b), except for those of canteen employees. Control measures comprised cohort isolation of symptomatic persons, exclusion of norovirus-positive canteen employees from work and disinfection of the canteen's kitchen.

**Conclusions:**

Our investigation indicated that consumption of norovirus-contaminated salad caused the peak of the outbreak on 7-9 January. Strict personal hygiene and proper disinfection of environmental surfaces remain crucial to prevent norovirus transmission.

## Background

Noroviruses are highly infectious pathogens that can cause relatively severe disease including vomiting and diarrhoea with acute onset. Symptoms usually resolve within 2 or 3 days. The incubation period ranges from 6-48 hours. Infections typically peak during the colder months [1,2]. Noroviruses are shed in high concentrations in faeces or vomit of infected persons. Their infectious dose is low [3]. The main mode of transmission is person-to-person [4], either faecal-orally or through aerosolised vomit. Indirect transmission via contaminated food, water, or environmental surfaces may also occur [5].

Approximately 50% of all gastroenteritis outbreaks in developed countries are caused by norovirus [1]. Globally, genotype (G) II-4 has been the predominant strain in outbreaks [6-9]. Outbreaks are frequently noticed in institutions such as hospitals, homes for the elderly and on cruise ships [10-12]. Eating food in restaurants, canteens or at catered events has also been related to norovirus outbreaks [13]. However, only few norovirus outbreak investigations which identified food items as potential vehicles of norovirus transmission through an analytical epidemiological study have been published [14-19]. Furthermore, potentially low norovirus concentrations in incriminated food items or environmental samples make detection difficult, due to a lack of sensitive and reliable testing methods [20,21].

In Germany, norovirus has been the most frequently reported pathogen causing an illness since 2004. In 2008, 80% of all reported outbreaks were caused by norovirus, and 59% of all reported norovirus infections occurred in clusters [22].

On 8 January 2009, the Bundeswehr Medical Service (German Armed Forces) reported an outbreak of acute gastroenteritis on a military base in Leipzig to the Robert Koch Institute. This specific military base had 815 members, and five divisions were stationed there. A total of 36 persons had fallen ill on 7 and 8 January with vomiting and diarrhoea. Food from the military base's canteen was suspected as the vehicle of infection. Norovirus was assumed to be the infectious agent due to the reported typical symptoms and the sudden onset of disease.

An outbreak investigation was initiated on 8 January to describe the outbreak's extent, verify norovirus as the causative pathogen, identify mode of transmission and source of infection, and to implement infection control measures to prevent further cases.

## Methods

### Descriptive epidemiology

We collected information on demographics, clinical symptoms, disease onset, and division memberships of persons with acute gastroenteritis. All members from the military base are obliged to visit a doctor of the Medical Service if ill. For this study, all persons with diarrhoea or vomiting between 24 December 2008 and 3 February 2009 were retrospectively and prospectively recorded. Data was entered in EXCEL (Microsoft 2003) and analysed using Stata (Stata Statistical Software: Release 10, Texas, USA). We described illnesses by onset of disease, age, sex and clinical symptoms, and calculated division-specific attack rates (AR).

### Cohort study

In order to test the hypothesis that food items served in the canteen were the vehicles of infection we conducted a retrospective cohort study. Through the head of the division, we distributed a self-administered standardised questionnaire (see Additional file 1) on 9 January to all 247 members of the headquarters company and informed about the purpose of the study. All potential study participants were ≥ 18 years old. Study participation was voluntary. We assumed implicit informed consent when a completed questionnaire was returned. The headquarters company was selected for the cohort study because it was the only division entirely present at the military base on the day of questionnaire distribution and because it was representative of all military base members regarding age and sex. The questionnaire included information on symptoms, date and time of symptom onset, and on the following possible exposures: food consumption on 5-7 January and contact to ill persons or to vomit. This time period was chosen as most likely to explain symptom onset on 5-9 January assuming an incubation period of 6-48 hours. Exposures on 3-4 January were not included in the exposure questionnaire as this was the final weekend of the Christmas holidays during which only few members were present at the military base.

Cases were defined as acute onset of diarrhoea or vomiting in a military base member between 5 and 9 January 2009, without detection of a pathogen other than norovirus in stools. We used EpiData software (EpiData Association, Odense, Denmark) for data entry and Stata (Stata Statistical Software: Release 10, Texas, USA) for data analysis.

For each exposure, we calculated the relative risk (RR) of becoming a case using the chi-square test and the attributable fraction (AF, only if RR > 1). Identified risk factors for illness and protective factors with a P value < 0.2 in the bivariable analysis were included in a multivariable logistic regression model to assess independent association with illness. In a stepwise backward procedure, we excluded exposures with P > 0.1 from the model. The exposure variable "contact to ill persons or to vomit during the incubation period" was consistently kept in the model to adjust for possible person-to-person transmission.

In order to assess possible dose-response relationships for food items independently associated with disease, we calculated AR and performed the chi-square test for trend.

### Laboratory analyses

#### Human stool samples

We collected stool samples from persons with acute gastroenteritis and from all canteen employees. Stool samples were tested for norovirus and, if negative, for *Salmonella, Shigella, Campylobacter, Yersinia*, *Staphylococcus aureus, Bacillus cereus, Clostridium perfringens*, enterohaemorrhagic *Escherichia coli *(EHEC), adeno- and rotavirus. For norovirus detection, stool samples were ten-fold diluted in phosphate-buffered saline (PBS, pH 7.2) and centrifuged for 1 minute at 13,500 × g. Viral nucleic acid was extracted from 140 μl of supernatant using QIAamp Viral RNA Mini Kit (Qiagen, Germany) according to manufacturer's instruction.

#### Environmental swabs

On 9 January, we sampled various surfaces of the military base's Medical Clinic facilities and cohort isolation rooms, the mail administrating centre, the canteen's kitchen, as well as the living and working areas. Using sterile swabs (VWR, Germany) moistened in PBS, 100 cm^2 ^of each environmental surface were wiped as described elsewhere [23]. Viral RNA from environmental samples was extracted by direct elution of the swab in the RNA-lysis buffer of the QIAamp Viral RNA Mini Kit (Qiagen, Germany) and processed further according to manufacturer's instruction.

#### Food items

At German military base canteens, meal items are routinely sampled and samples are kept for 48 hours. According to the instructions, sampling is performed between preparation and distribution of food. The specific food items served and sampled in the canteen on 6 and 7 January were tested for norovirus if the results from the analytical study suggested an independent association with disease. Samples from food served on 5 January had already been discarded. For testing, we used a method adapted from a previously reported elution-precipitation protocol [24]. Viscous food samples such as dressings and gravies were analysed using an ultrafiltration protocol [25]. Viral nucleic acid was extracted as described above.

#### Molecular detection of norovirus

The extracted RNA was tested for norovirus by real-time reverse-transcription polymerase chain reaction (RT-PCR) [26] in an ABI PRISM 7500 cycler using the Quantitect Probe RT-PCR Kit (Qiagen, Hilden, Germany) permitting the differentiation between genogroup (G) I and II. Positive samples were additionally amplified by RT-PCR in the open reading frame 1 (ORF1) according to [27], and in the junction gene region between ORF1 and ORF2 using the One-Step RT-PCR Kit (Qiagen, Hilden, Germany). As only GII was detected in this outbreak, GII specific primers used for the amplification within the ORF1/2 junction were NV 107c (sense, 5'- AICCIATGTTYAGITGGATG, nt position 5007 to 5026) and NV 156 (antisense, 5' - ACCKGCATAACCATTRTACAT, nt position 5387 to 5367) for the first round, and NV107c and NV300/II (antisense, 5'- CYAGGKGCYTGIACAAARTT, nt position 5266 to 5247, all positions according to GenBank AY485642) for the second round PCR. The amplicons were separated on ethidium bromide-stained 1.5% agarose gels and visualised under UV light.

#### Sequence analysis

The amplicons of the ORF1 (213 bp) and the ORF1/2 junction region (215 bp) considered for sequence analysis were purified using the Qiaquick DNA purification kit (Qiagen, Hilden, Germany). Amplicons of the ORF 1/2 junction region were directly sequenced using the amplification primers of the second round PCR. Amplicons of the ORF1 region were sequenced using generic norovirus primers MON431 and MON432 [27] or cloned into pCR4-TOPO vector using the TOPO TA Cloning Kit for Sequencing (Invitrogen, Karlsruhe, Germany). Cloned fragments were sequenced using the M13 Forward primer (Invitrogen, Karlsruhe, Germany) in an ABI 3730 DNA Analyzer (Applied Biosystems, Carlsbad, California, USA). Sequence alignment to prototype sequences drawn from GenBank was performed using CLUSTAL W and the NEIGHBOR JOINING and DNADIST program of the Phylogeny Interference Package (PHYLIP) version 3.57c [28] or the MegAlign module of the DNASTAR software package (Lasergene, Madison, USA).

## Results

### Descriptive epidemiology

Overall, 104 of 815 military base members (AR 12.8%) fell ill with vomiting or diarrhoea between 24 December 2008 and 3 February 2009. Three norovirus-negative persons shed rotavirus, *Campylobacter *and EHEC in their stools, see laboratory results.

**Figure 1:**
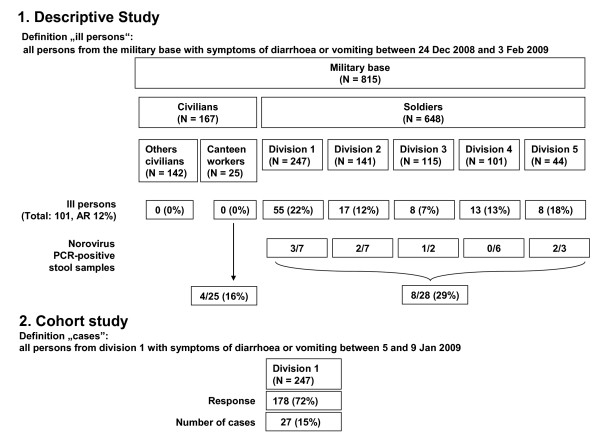
**Overview of persons participating in the descriptive and cohort study**. Norovirus-outbreak at a military base, Germany, 2009.

**Figure 2:**
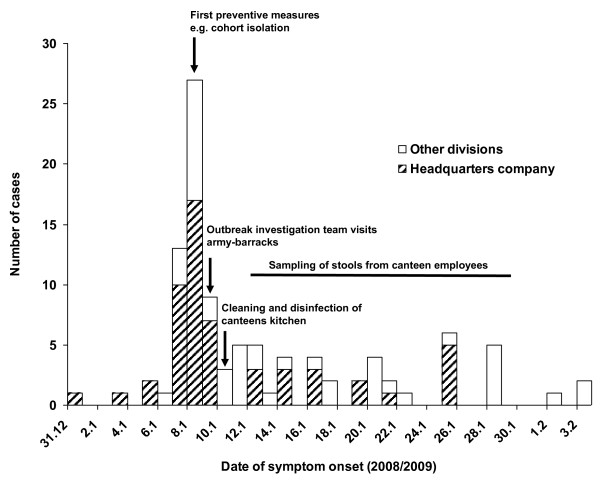
**Gastroenteric illnesses by onset of disease and division**. Norovirus-outbreak at a military base, Germany, 2009 (N = 101).

In the following, we describe the 101 ill persons without detection of any other pathogen than norovirus (Figure 1). The first person fell ill on 31 December, a second one on 3 January, and two more on 5 January. The number of ill persons peaked during 7-9 January (n = 49) (Figure 2). Zero to six persons (median 1) per day fell ill between 10 January and 3 February. Hereafter, the outbreak ceased. The ill persons' median age was 22 years (range: 18-64). Of 99 ill persons with available information on sex, 92.9% were male. A total of 34 (33.7%) ill persons suffered from both, vomiting and diarrhoea. Vomiting only was reported by 32 (31.7%) and diarrhoea by 35 (34.7%). Division-specific AR were highest in the headquarters company (55 of 247, 22.3%) and among staff of the Medical Clinic (8 of 44, 18.2%).

### Cohort study

Overall, 178 of 247 (72.1%) persons from the headquarters company completed the questionnaire. Among those, 160 stated the date of questionnaire completion: 31.9% completed it before 12 January (within seven days after the earliest date of food consumption we queried in our questionnaire), 64.4% before 15 January (within seven days after the latest date of food consumption queried) and further 3.8% until 22 January. Information on non-responders was not available.

Overall, 27 persons met the case definition (AR 15.2%). Two persons fell ill on 5 January, eight on 7 January, 11 on 8 January and six on 9 January. In cases with available information, the median duration of illness was two days (range: one to four, n = 21), the median duration of sick leave was four days (range: zero to nine, n = 16) and four of eighteen were hospitalized (22%). Cases and non-cases did not differ with regard to age and sex (both median 24 years, range: 18-58, and 96% male).

Overall, 25 of 133 (18.8%) persons who ate any meal at the military base's canteen and two of 45 (4.4%) persons who did not eat there during 5-7 January met the case definition (RR: 4.3; 95% CI: 1.1-17.4; P < 0.05). In bivariable analyses, persons consuming any lunch during 5-7 January at the canteen had an increased risk of disease compared to those who did not. Eating breakfast or eating dinner at the canteen, contact to ill persons or vomit during the incubation period or staying at the military base overnight were not associated with disease (Table 1).

**Table 1:** Bivariable analysis of risk factors*.

	Exposed			Non-exposed				Bivariable		
Exposure	Total	Cases	AR%	Total	Cases	AR%	AF%	RR	95% CI	P
**Eating any meal at canteen**	**133**	**25**	**18.8**	**45**	**2**	**4.4**	**76.4**	**4.3**	**1.1-17.4**	**0.02**
Eating any breakfast at canteen	62	9	14.5	116	18	15.5	-6.9	0.9	0.5-2.0	0.88
**Eating any lunch at canteen**	**125**	**25**	**20.0**	**53**	**2**	**3.8**	**81.2**	**5.2**	**1.3-21.3**	**0.01**
Eating any dinner at canteen	49	9	18.4	129	18	14.0	24.1	1.3	0.6-2.7	0.49
Contact to ill persons or vomit	35	8	22.9	143	19	13.3	41.9	1.7	0.8-3.6	0.17
Staying at military base overnight	67	11	16.4	108	16	14.8	9.8	1.1	0.6-2.2	0.78

AR = attack rate, RR = relative risk, CI = confidence intervall, AF = attributable fraction

*Norovirus outbreak at a military base, Germany, 2009. Retrospective cohort study among members of the headquarters company with risk of infection between 5-7 January (n = 178). Exposures with P < 0.05 in bold.

Hence, we performed a day-specific analysis restricted to persons who ate lunch at the military base's canteen on any particular day during 5-7 January to identify food items consumption of which was associated with becoming a case. The inclusion of cases was limited to those having consumed food in the canteen 6-48 hours before disease onset. We excluded cases without information on the time of disease onset.

None of the 113 respondents potentially exposed during lunch on 5 January met the case definition.

During lunch on 6 January, 106 of 178 (59.6%) respondents were potentially exposed. Among those, 11 (AR 10.4%) met the case definition. Eating salad from a self-service salad buffet was associated with higher risk of disease in bivariable and multivariable analysis (Table 2).

**Table 2:** Bivariable and final model of multivariable analysis of risk factors, 6 January 2008*.

	Exposed			Non-exposed				Bivariable			Multivariable		
Exposure	Total	Cases	AR%	Total	Cases	AR%	AF/PF %	RR	95% CI	P	aOR	95% CI	P
**Salad bar**	**50**	**9**	**18.0**	**56**	**2**	**3.6**	**80.2**	**5.0**	**1.1-22.2**	**0.02**	**8.1**	**1.5-45.4**	**0.02**
**Potatoes**	**39**	**1**	**2.6**	**57**	**10**	**17.5**	**585.2**	**0.2**	**0.0-1.1**	**0.02**			
**Schnitzel**	**49**	**1**	**2.0**	**51**	**10**	**19.6**	**861.3**	**0.1**	**0.0-0.8**	**0.01**	0.1	0.0-1.4	0.09
Pasta	48	8	16.7	55	3	5.5	67.3	3.1	0.9-10.9	0.07	1.8	0.3-12.0	0.58
Fish	7	2	28.6	90	9	10.0	65.0	2.9	0.8-10.1	0.14			
Vegetables	26	1	3.9	68	10	14.7	282.1	0.3	0.0-1.9	0.14			
Contact to ill persons or vomit	28	1	3.6	78	10	12.8	259.1	0.3	0.0-2.1	0.17	0.2	0.0-1.8	0.15
Yoghurt	17	0	0.0	78	11	14.1	NC	0.0	**∞-∞**	0.10			

AR = attack rate, RR = crude relative risk, CI = confidence intervall, AF/PF = attributable/preventable fraction, aOR = adjusted odds ratio

* Norovirus outbreak at a military base, Germany, 2009. Retrospective cohort study among members of the headquarters company with risk of infection during lunch on 6 January (n = 106). Exposures only listed if P < 0.2, and if P < 0.05 in bold.

During lunch on 7 January, 108 of 178 (60.1%) respondents were potentially exposed. Of those, 19 (AR 17.6%) met the case definition. In bivariable and multivariable analysis, consumption of salad was again associated with increased risk of disease (Table 3).

**Table 3:** Bivariable and final model of multivariable analysis of risk factors, 7 January 2008*.

	Exposed			Non-exposed				Bivariable			Multivariable		
Exposure	Total	Cases	AR%	Total	Cases	AR%	AF%	RR	95% CI	P	aOR	95% CI	P
**Salad bar**	**48**	**14**	**29.2**	**57**	**2**	**3.5**	**88.0**	**8.3**	**2.0-34.8**	**0.00**	**11.6**	**2.5-54.5**	**0.00**
Contact to ill persons or vomit	26	4	15.4	79	12	15.2	1.2	1.0	0.4-2.9	0.98	1.3	0.7-0.3	0.71

AR = attack rate, RR = crude relative risk, CI = confidence interval, AF = attributable fraction, aOR = adjusted odds ratio

* Norovirus outbreak at a military base, Germany, 2009. Retrospective cohort study among members of the headquarters company with risk of infection during lunch on 7 January (n = 108). Exposures only listed if P < 0.2, and if P < 0.05 in bold.

We assessed a possible dose-response relationship between salad consumption and disease by including only persons in our analysis who ate lunch at the canteen on all days between 5 and 7 January. The risk of disease increased with the number of days on which salad was consumed (Table 4).

**Table 4:** Dose-response-relationship between number of days of salad consumption and risk of disease*.

Salad consumption	Total	Cases	AR%	OR	95% CI	P
None	44	2	4.5	reference		
On any one day	14	2	14.3	3.5	0.4-28.8	0.21
**On any two days**	**11**	**3**	27.3	**7.9**	**1.0-62.2**	**0.02**
**On all three days**	**26**	**11**	42.3	**15.4**	**2.4-97.5**	**0.00**

AR = attack rate, OR = odds ratio, CI = confidence interval

*Norovirus-outbreak at a military base, Germany, 2009. Retrospective cohort study among members of the headquarters company eating every lunch at the military base's canteen between 5-7 January (n = 95).

Within the entire cohort, persons eating salad (21 of 75, AR: 28.0%) had a 4.8-times higher risk (95% CI: 2.1-11.4; P < 0.001) of being a case than persons not consuming salad (6 of 103, AR: 5.8%). Among persons who had lunch in the canteen on 6 and 7 January (n = 119), the AR among salad consumers (16 of 65, 24.6%) was 4.4-times higher (95% CI: 1.4-14.4; P < 0.01) than among non-salad eaters (3 of 54, 5.6%).

### Laboratory results

#### Stool samples

Overall, 8 of 28 (28.6%) tested stool samples from ill persons were norovirus genogroup II-PCR-positive. Samples were taken between 12 January and 3 February, a median of 4 days after onset of illness (range 1-10 days). Only one sample per person was taken into consideration. Sequencing of five randomly selected specimens revealed an identical subtype (GII.4-2006b) and identical nucleotide sequences in the polymerase and the ORF1/2 gene region, respectively. Stool specimens from one ill person, respectively, tested norovirus-negative but positive for rotavirus, *Campylobacter *or EHEC.

**Figure 3:**
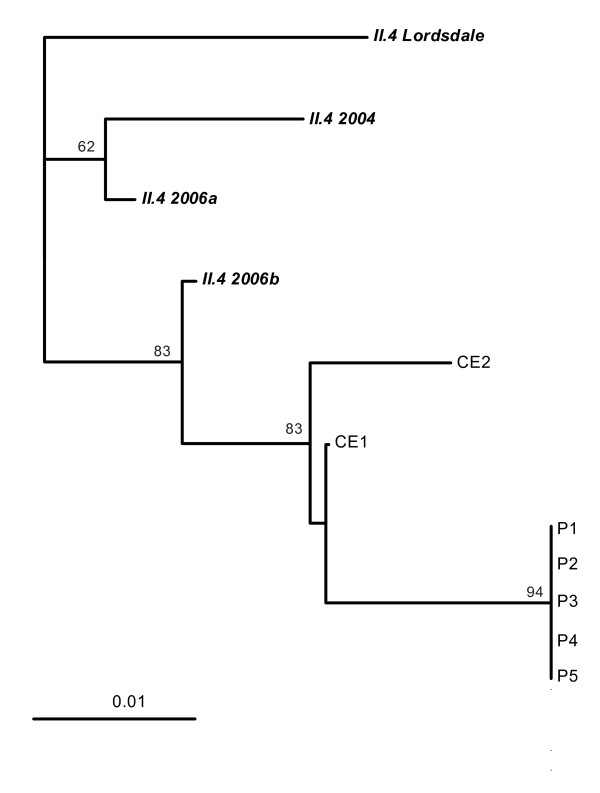
**Neighbour-joining tree of 215 bp amplificates of the ORF1/2 junction of norovirus**. P1 to P5 are sequences from stool specimens of patients and CE1 and CE2 from canteen employees. Prototype sequences in bold and italics are from GenBank: II.4 Lordsdale, X86557; II.4 2004 DenHaag54, EF126962; II.4 2006a Terneuzen70, EF126964; II.4 2006b Nijmegen115, EF126966. Bootstrap values > 60% are indicated. The scale represents nucleotide substitutions per site.

Stool samples of 4 of 25 canteen employees (16%) taken during 12-29 January tested norovirus-positive. Two of the norovirus-positive persons had worked in the canteen's kitchen between 5 and 7 January. One of them reported to have suffered from nausea with onset on 5 January. She had continued working through 6 January. The other reported not to have had any symptoms. She had cleaned the salad bar on 6 and 7 January. Norovirus sequences found in stool samples of these two persons belonged both to subtype GII.4-2006b but differed between 1.4% and 2.4% from those detected in ill persons (Figure 3). Nucleotide sequences of the ORF1/2 gene region from one ill person and two canteen employees were submitted to GenBank (accession numbers GU017736, GU017737, and GU017738).

The other two canteen employees shedding norovirus did not work at the canteen in January 2009 at all. EHEC was isolated in one other symptomatic canteen employee. All other stool samples from canteen employees tested negative for bacterial or viral pathogens.

#### Environmental swabs

Norovirus was detected in 6 of 55 (10.9%) environmental samples taken on 9 January. Positive swabs stemmed from the mail administrating centre (3 of 8 swabs positive: mail counter, mail collection box, doorknob), the living or working areas (2 of 16: computer keyboard, toilet flush), and the cohort isolation rooms (1 of 12: doorknob). Norovirus was neither found in any swabs from the kitchen (0 of 15) nor from the canteen employees' toilets (0 of 4). Surfaces from the salad bar and the dining hall were not sampled. Since PCR products were mostly too weak for confirmation by sequencing, only three environmental samples (computer keyboard, mail collection box and doorknob from the mail centre) were sequenced. They belonged to norovirus subtype II.4-2006b and were of identical sequence to the ill persons' clinical samples.

#### Food items

None of the 33 tested food items was norovirus PCR-positive. Investigated food items included salads and salad dressings.

### Infection control measures

On 8 January, the heads of all five military divisions were briefed on the outbreak and on preventive measures. Ill persons were either cohort isolated in assigned rooms or went on sick leave for one week. Hand disinfectants were distributed to ill persons. Healthy persons were sent home for two days (8 and 9 January) to interrupt the outbreak. Specific soldiers were designated and instructed for cleaning and disinfection of areas contaminated with vomit and faeces.

The canteens' kitchen was routinely cleaned on 8 and 9 January. On 9 January, the military unit responsible for hygiene inspected the kitchen. Cleaning and disinfection was performed on 10 January using virucidal agents. Canteen employees with positive stool samples were excluded from work until tested negative for the respective infectious agent.

## Discussion

We investigated an outbreak of acute gastroenteritis that affected 12% of the members of a military base and that was caused by norovirus of subtype II.4-2006b. The outbreak lasted for 5 weeks. Half of the illnesses occurred within 3 days between 7 and 9 January, strongly indicating a point source of infection. Results of the cohort study suggested food-borne transmission for most of these cases. Eating any meal, having any lunch, and consuming salad from a self-service buffet at the military base's canteen were all highly associated with disease. Frequency of salad consumption correlated positively with risk of disease. Of the 27 cases identified in the cohort study, 25 had eaten lunch and 21 had eaten salad at the canteen supporting the hypothesis that infections were acquired during canteen visits. Person-to-person transmission was tested for but not identified as risk factor for becoming a case. However, for the time period before and after 5-9 January, both person-to-person and environmental-to-person transmission is plausible. Norovirus outbreaks with initial food-borne transmission further propagated by person-to-person or environmental-to-person transmission have been reported [1,29].

Our findings of a causal role for salad in this outbreak are consistent with other studies in which salad had been involved in norovirus transmission [1,17,19,30]. The salad which was mainly eaten raw might have been contaminated during cultivation, harvest, or by food handlers during preparation [21]. Alternatively, persons shedding norovirus could have contaminated food items or surfaces while serving themselves at the salad bar [1,13]. Possible cross-contamination at salad buffets with self-service might be prevented by offering already prepared and portioned salads in bowls.

All analysed food samples including salad were tested norovirus PCR-negative. Reasons for the norovirus PCR-negative food samples could be the presence of substances inhibiting molecular detection such as phenolics and polysaccharides, low virus concentration, or a heterogeneous distribution of viruses in the food. Other studies confirm that norovirus detection in food items remains challenging [31,32].

Norovirus genome sequence analysis suggests that all ill persons belonged to the same outbreak. However, the specific source of contamination for the salad, the likely vehicle of infection, remains unclear. While norovirus genome sequences from ill persons and environmental swabs were identical, those from canteen employees differed slightly.

Therefore it its unlikely that canteen employees contaminated the salad. Since norovirus infections peak every winter, occurrence of norovirus-infected persons in the military base due to virus circulation in the general population is plausible. However, persons shedding several different norovirus strains at the same time have been reported [33]. Hence, the two norovirus-positive canteen employees might have been shedding the outbreak norovirus-strain without detection when samples were taken one to four weeks after the start of the outbreak.

Norovirus-contaminated environmental surfaces might have played a role in transmission. Their implication in virus transmission has been documented in other studies [34-38]. In our investigation, surface contamination was not restricted to the immediate environment of ill persons which highlights the difficulties in identifying and properly disinfecting all possible norovirus-contaminated environmental surfaces during outbreaks. We were able to detect norovirus on various surfaces including a mail delivery box and a computer keyboard indicating widespread environmental contamination. This is not surprising since many individuals experienced symptoms of norovirus infection while in the military base. Noroviruses are able to persist in an infective state on environmental surfaces for several days after initial contamination [39], e.g. on computers and telephones [40]. However, since the members of the five divisions worked at different buildings and assumingly not everyone went e.g. to the mail administrating centre, the canteen is the more likely location of norovirus transmission for the majority of cases.

The date of sampling might explain why environmental swabs from the canteens' kitchen were norovirus PCR-negative. Samples were drawn on 9 January. If the contamination occurred on 6 or 7 January, norovirus could have been already removed on 9 January due to the daily regular cleaning.

Cases might have remembered information on food consumption better than non-cases. Since we provided the list of served food items in the questionnaire and most participants completed the questionnaire within ten days after possible exposure, we assume the impact of recall bias as minor. Possibly, the low proportion of norovirus PCR-positive cases was partly due to the ordinance of sick leave of one week for ill persons in contrast to special leave of two days for healthy persons. This might have tempted some soldiers to pretend being ill resulting in an overestimation of case numbers. However, this potential non-differential misclassification would probably have led to an underestimation of the true association between salad consumption and risk of disease. Another reason for the low proportion of norovirus PCR-positive cases could be that people did not shed virus anymore at the time of sampling.

Generally, military bases provide good conditions for epidemiological investigations: the source population is distinct, soldiers are instructed to visit the same doctor for medical advice, food samples are collected routinely from each meal and are kept for 48 hours, and the military is well equipped for timely investigations, which is especially helpful in food-borne disease outbreaks.

Early implemented preventive measures like cohort isolation and sick leave for ill persons, special leave for healthy military base members, distribution of hand disinfectants, cleaning and disinfection of the Medical Clinic and the canteens' kitchen as well as identification and exclusion of norovirus PCR-positive canteen employees from work may have successfully averted further cases.

In addition to recommendations generally applicable to outbreaks of acute gastroenteritis such as timely and sustained implementation of preventive measures, we derive the following recommendations from the experience in this outbreak. Surfaces that are touched by many people, such as doorknobs or computer equipment, should be cleaned and disinfected daily or more often than usual to avert environmental-to-person transmission.

Protective measures should be communicated in oral and written forms. Posters displayed clearly visible in buildings with many passers-by should prompt military base members and visitors to wash their hands with water and soap and use disinfectants after toilet visits and before food consumption. Adequate hand disinfectants should be placed next to sinks for both healthy and ill persons.

As we found asymptomatic norovirus-shedding canteen employees, the implications of hand washing and disinfection before handling food or touching surfaces used for food preparation, especially after going to the toilet, should be communicated more frequently to staff members. If canteen employees fall ill, they must stop working immediately. Resumption of work should be earliest two days after remission of symptoms.

Sequence analysis of predominant norovirus genotypes like GII.4 should be performed in order to identify chains of infection. Further research on methods for norovirus detection in food is needed.

## Conclusions

Our investigation indicated that consumption of norovirus-contaminated salad caused the peak of the outbreak on 7-9 January. Strict personal hygiene and proper disinfection of environmental surfaces remain crucial to prevent norovirus transmission.

The timely initiated analytical epidemiological investigation proved valuable to identify the likely vehicle of infection and to guide targeted intervention measures.

## Competing interests

The authors declare that they have no competing interests.

## Authors' contributions

MW participated in the design of the study, drafted questionnaires, performed data entry, epidemiological analysis and interpretation of the data, participated in coordinating the study and wrote the manuscript draft. KSc carried out the virological analysis, interpreted the laboratory results and wrote the manuscript draft. SN participated in the design of the study, drafted questionnaires, took part in the analysis of the data and contributed to the manuscript. SD participated in molecular analysis of human stool samples. LE has made contributions to conception and design and organizing the acquisition of data. CF was involved in study planning, data analysis and contributed to manuscript writing.

RG carried out microbiological and virological analysis and contributed to the manuscript. MH performed sequencing and phylogenetic analysis of norovirus from human stool samples. RJ was involved in study coordination, analysis and interpretation of the study, and contributed to the manuscript. GK was involved in analysis and interpretation of the study regarding laboratory aspects. JK participated in the design of the study, assisted the outbreak investigation and revised the manuscript critically. JS participated in coordinating the study and revised the manuscript critically. UT participated in coordinating the study in the field and contributed to manuscript writing. KSt was involved in study conception, design, and coordination of the study, data analysis and contributed to manuscript writing. HB was involved in study conception, design, and coordination, data entry, data analysis, data interpretation, and manuscript writing. All authors read and approved the final manuscript.

## Pre-publication history

The pre-publication history for this paper can be accessed here:

http://www.biomedcentral.com/1471-2334/10/30/prepub

## Supplementary Material

Additional file 1 **Questionnaire distributed to members of the headquarters company, norovirus-outbreak at a military base, Germany, 2009**. The questions focus on potential risk factors for norovirus-outbreaks like food items eaten at the canteen and contact to ill persons or vomit. Click here for file
