# Supplementary material for: Food-borne norovirus-outbreak at a military base, Germany, 2009
Source: BMC Infect Dis. 2010 Feb 17;10:30. doi: 10.1186/1471-2334-10-30 (PMC2831023; doi:10.1186/1471-2334-10-30)
Supplement: Additional file 1 — Questionnaire distributed to members of the headquarters company, norovirus-outbreak at a military base, Germany, 2009. The questions focus on potential risk factors for norovirus-outbreaks like food items eaten at the canteen and contact to ill persons or vomit. [file 1471-2334-10-30-S1.DOC]

**Norovirus-outbreak at a military base, Germany, 2009**

**Interviewer: __________________________ Date: __ __/__ __/__ __**

###

### **Date of birth (DD/MM/YYYY): _____/______/_______ Sex** male female

### **Military grade: _____________________ Division/Subdivision: __________________ /__________________**

Overnight at military base:  yes  no

If yes, accommodation: Building ____________ Floor ____________ Room ____________________

Number of persons sharing the room: ______

Position: Soldier

In Training

Canteen worker

Cleaning personal

Other position _______________________________

1. Did you suffer from gastro-enteric disease since 31 December 2008?

yes no don’t know

If yes, when was the onset of symptoms? **/**____**/**____**/**____**/** Date ________ Time (hour)

How many days lasted the symptoms? ____ days Symptoms still present

If yes, which symptoms did you suffer from?

Diarrohea yes no don’t know

How many times within 24 hours at worst day: ___ x

Vomiting yes no don’t know

How many times within 24 hours at worst day: ___ x

Where did you vomit (please list specific locations of vomiting)? _________________________________________________________________

_________________________________________________________________

Sickness yes no don’t know

Fever (>38.5°C) yes no don’t know

Tummy ache yes no don’t know

Shivering yes no don’t know

Head ache yes no don’t know

Muscle/joint pain yes no don’t know

Acute onset of disease

yes no don’t know

Blood in stool yes no don’t know

2. Did you see a medical doctor because of your symptoms?

Yes No Don’t know

If yes, were you put on sick leave?  yes no  don’t know

If you were on sick leave, for how many days? ______

3. Was your stool sampled?

Yes No Don’t know

4. Were you hospitalized because of your symptoms?

Yes No Don’t know

If yes, for how many days? ____ days

5. Do you have any idea, why you got sick?

6. Is a person sharing your room sick (gastro-enteric symptoms)?

Yes No Don’t know

If yes, was onset of symptoms of this/these person/s before or after your own onset of symptoms?

Person 1:  before  after  I was not sick myself

Person 2:  before  after  I was not sick myself

Person 3:  before  after  I was not sick myself

7. Did you have contact zu sick persons outside of the military-base (gastro-enteric symptoms) ?

Yes No Don’t know

If yes, was onset of symptoms of this/these person/s before or after your own onset of symptoms?

Person 1:  before  after  I was not sick myself

Person 2:  before  after  I was not sick myself

Person 3:  before  after  I was not sick myself

8. Have you seen a person vomit?

Yes No Don’t know

9. Have you shared a room with a person who vomitted?

Yes No Don’t know

10. Have you visited a bathroom with signs of vomits on the toilets or in the sinks?

Yes No Don’t know

11. Did a person vomit while you were sitting with him/her at the table?

Yes No Don’t know

12. Did you remove vomits during your stay at the military-base?

Yes No Don’t know

13. Did you help someone, who vomitted?

Yes No Don’t know

14. Food: Which of the following meals did you consume in the military-bases canteen?

**(Specific food items in the annex)**

**Monday, 5. January 2009**

Breakfast

Yes No Don’t know

Lunch

Yes No Don’t know

Dinner

Yes No Don’t know

**Tuesday, 6. January 2009**

Breakfast

Yes No Don’t know

Lunch

Yes No Don’t know

Dinner

Yes No Don’t know

**Wednesday, 7. January 2009**

Breakfast

Yes No Don’t know

Lunch

Yes No Don’t know

Dinner

Yes No Don’t know

15. Did you drink water from the sink during your stay in the military-base?

Yes No Don’t know

Number of 0,2l glasses/day? ____

16. Which of the following drinks and food items did you consume during your stay in the military-base?

- Mineral water Yes No Don’t know
- Fruit juices with mineral water Yes No Don’t know
- Fruit juices Yes No Don’t know
- Ice cubes in drinks Yes No Don’t know
- Salads (uncooked) Yes No Don’t know
- Fresh fruits Yes No Don’t know
- Smoked fish Yes No Don’t know
- Sea food Yes No Don’t know

17. Comments:

**Monday, 5. January 2009**

**Breakfast**

Frühstücksbrötchensortiment Yes  No  Don’t know

Mehrkorn-, Zwiebel-, Knaeckebrot, Pumpernickel, Zwieback

Yes  No  Don’t know

Deli-Leberwurst Yes  No  Don’t know

Hinterschinken Yes  No  Don’t know

Salami Yes  No  Don’t know

Gouda Yes  No  Don’t know

Tilsiter Yes  No  Don’t know

Suesser Aufstrich Yes  No  Don’t know

Konfitüren Yes  No  Don’t know

Sirup Yes  No  Don’t know

Schokoladencreme Yes  No  Don’t know

Butter Yes  No  Don’t know

Halbfettmargarine Yes  No  Don’t know

Frischkäse Yes  No  Don’t know

Zerealien Yes  No  Don’t know

Quarkzubereitung Yes  No  Don’t know

Joghurtzubereitung Yes  No  Don’t know

Gekochtes Ei Yes  No  Don’t know

Knabbergemuese Yes  No  Don’t know

Obst Yes  No  Don’t know

**Lunch, please state approximate amount of food**

**normal portion**  **not finished**  **got an extra portion**

Lauchcremesuppe Yes  No  Don’t know

Schweinefleischpfanne „Mexiko“ Yes  No  Don’t know

Kraeuterreis Yes  No  Don’t know

Feld-, Tomaten-, Gurken-, Weißkraut-, Paprika-, Eisbergsalat

Yes  No  Don’t know

Salatdressings, Croutons Yes  No  Don’t know

Birnen-Joghurt-Quarkspeise Yes  No  Don’t know

Zwiebelquark mit Schnittlauch, Leinoelbutter, Leberwurst

Yes  No  Don’t know

Pertersilienkartoffeln Yes  No  Don’t know

Pfirsich mit Himbeersauce Yes  No  Don’t know

Thunfischsalat auf Toastbrot Yes  No  Don’t know

Nudeleintopf mit Gefluegelfleisch Yes  No  Don’t know

Karamellpudding Yes  No  Don’t know

Quarkkeulchen mit Vanillesauce Yes  No  Don’t know

Penne all’arrabbiata (scharfe Sauce) Yes  No  Don’t know

Mousse au chocolat mit Kokosflocken Yes  No  Don’t know

**Dinner**

Butter Yes No Don’t know

Halbfettmargarine Yes No Don’t know

Joghurtbutter Yes No Don’t know

Frischkäse Yes No Don’t know

Brotsortiment Yes No Don’t know

Butterkaese Yes  No  Don’t know

Gouda Yes  No  Don’t know

Quarkjoghurt Yes  No  Don’t know

Stückobst Yes  No  Don’t know

Salat aus Gurken, Tomaten, Radieschen, Mais Yes  No  Don’t know

Kartoffelecken mit Dip Yes  No  Don’t know

Truthahnbierschinken Yes  No  Don’t know

Schinkenpaste Yes  No  Don’t know

Truthahnfleischkaese Yes  No  Don’t know

Kasselerbraten kalt Yes  No  Don’t know

Heringssalat rot Yes  No  Don’t know

Krabbensalat Yes  No  Don’t know

**Tuesday 6. January 2009**

**Breakfast**

Frühstücksbrötchensortiment Yes No Don’t know

Zwiebelbrot- und Malfabrot, Zwieback Yes No Don’t know

Paprikalyoner Yes No Don’t know

Rohe Knoblauchwurst Yes No Don’t know

Gekochter Hinterschinken Yes No Don’t know

Hackfleischbällchen Yes No Don’t know

Schnittlauchkäse Yes No Don’t know

Glockenzeller Yes No Don’t know

Konfitüren Yes No Don’t know

Sirup Yes No Don’t know

Schokoladencreme Yes No Don’t know

Butter Yes No Don’t know

Halbfettmargarine Yes No Don’t know

Frischkäse Yes No Don’t know

Zerealien Yes No Don’t know

Quarkzubereitung Yes No Don’t know

Joghurtzubereitung Yes No Don’t know

Knabbergemüse Yes No Don’t know

Obst Yes No Don’t know

**Lunch, please state approximate amount of food**

**normal portion**  **not finished**  **got an extra portion**

Tomatenrahmsuppe mit Basilikum und Mozarella Toast Yes No Don’t know

Schweineschnitzel „Cordon Bleu“ Yes No Don’t know

Kartoffeln Yes No Don’t know

Leipziger Allerlei Yes No Don’t know

Tomaten-, Gurken-, Möhren, Rote Beete-, Eisbergsalat Yes No Don’t know

Salatdressings, Schinkenstreifen, Fetakäsewürfel Yes No Don’t know

Zigeunersalat mit Zwiebelbrot Yes No Don’t know

Wurstgulasch Yes No Don’t know

Makkaroni Yes No Don’t know

Reibekäse Yes No Don’t know

Joghurtzubereitung Yes No Don’t know

Fischroulade mit Gemüse Yes  No  Don’t know

Kräuterbechamelsauce Yes No Don’t know

Kartoffelpüree Yes No Don’t know

Tomaten-, Gurken-, Möhren, Rote Beete-, Eisbergsalat Yes No Don’t know

Salatdressings, Schinkenstreifen, Fetakäsewürfel Yes No Don’t know

gebackene Blumenkohlröschen Yes No Don’t know

Champignontasche Yes No Don’t know

Kartoffelecken Yes No Don’t know

Brokkoli mit Mandelsplittern Yes No Don’t know

Tomaten-, Gurken-, Radieschen-, Kopfsalat Yes No Don’t know

Dressingsortiment und Croutons Yes No Don’t know

**Dinner**

Butter Yes No Don’t know

Halbfettmargarine Yes No Don’t know

Joghurtbutter Yes No Don’t know

Frischkäse Yes No Don’t know

Brotsortiment Yes No Don’t know

Gouda Yes No Don’t know

Butterkäse Yes No Don’t know

Stückobst Yes No Don’t know

Gurken und Tomaten, frisch Yes No Don’t know

Pizzabaguette Salami Yes No Don’t know

Currywurst Yes No Don’t know

Katenrauchwurst Yes No Don’t know

Norddeutsche Mortadella Yes No Don’t know

Heringsfilet Yes No Don’t know

**Wednesday, 7 January 2009**

**Breakfast**

Frühstücksbrötchensortiment Yes No Don’t know

Roggenmisch- u. Leinsamenbrot, Pumpernickel,
Zwieback Yes No Don’t know

Paprikalyoner Yes No Don’t know

Schinkenpastete Yes No Don’t know

Truthahnfleischkäse Yes No Don’t know

Camembert Yes No Don’t know

Glockenzeller Yes No Don’t know

Konfitüren Yes No Don’t know

Sirup Yes No Don’t know

Schokoladencreme Yes No Don’t know

Butter Yes No Don’t know

Halbfettmargarine Yes No Don’t know

Frischkäse Yes No Don’t know

Zerealien Yes No Don’t know

Quarkzubereitung Yes No Don’t know

Joghurtzubereitung Yes No Don’t know

Knabbergemüse Yes No Don’t know

Obst Yes No Don’t know

Rührei Yes No Don’t know

**Lunch, please state approximate amount of food**

**normal portion**  **not finished**  **got an extra portion**

Kartoffelrahmsuppe mit Buttercroutons Yes No Don’t know

Rindersaftbraten Schweizer Art Yes No Don’t know

Salzkartoffeln Yes No Don’t know

Rosenkohl Yes No Don’t know

Kartoffel-, Gurken-, Weißkraut-, Eisbergsalat Yes No Don’t know

Dressingsortiment und Croutons Yes No Don’t know

Erdbeerpudding Yes No Don’t know

Kartoffelrahmsuppe mit Buttercroutons Yes No Don’t know

Schweinegulasch mit Gemüse Yes No Don’t know

Hörnchen Yes No Don’t know

Kartoffel-, Gurken-, Weißkraut-, Eisbergsalat Yes No Don’t know

Dressingsortiment und Croutons Yes No Don’t know

Obstsalat Tropic Yes No Don’t know

Heringssalat weiß mit Leinsamenbrot Yes No Don’t know

Putenbrust mit Bambussprossensauce Yes No Don’t know

Risotto Yes No Don’t know

Chinagemüse Yes No Don’t know

Kartoffel-, Gurken-, Weißkraut-, Eisbergsalat Yes No Don’t know

Dressingsortiment und Croutons Yes No Don’t know

Quark-Joghurtzubereitung Yes No Don’t know

Tomate Mozzarella mit Basilikum Yes No Don’t know

Blumenkohl Yes No Don’t know

Gemüsereis Yes No Don’t know

Tomaten, Bohnen-, Karotten-, Gurken-, Blattsalat Yes No Don’t know

Dressingsortiment und Croutons Yes No Don’t know

**Dinner** Butter Yes No Don’t know

Halbfettmargarine Yes No Don’t know

Joghurtbutter Yes No Don’t know

Frischkäse Yes No Don’t know

Brotsortiment Yes No Don’t know

Gouda Yes No Don’t know

Butterkäse Yes No Don’t know

Stückobst Yes No Don’t know

Gurken und Tomaten, frisch Yes No Don’t know

Grillhaxe Yes No Don’t know

Putensalami Yes No Don’t know

Katenrauchwurst Yes No Don’t know

Truthahnlyoner Yes No Don’t know

Seelachsersatz Yes No Don’t know
